# Schizothoracinae in Plateau River Networks: Drainage History, Polyploid Genome Evolution, Multi-Omics Evidence Chains, and Conservation Units

**DOI:** 10.3390/ani16071036

**Published:** 2026-03-28

**Authors:** Yongqing Cao, Ning Wang, Qiaomu Hu, Xiangyun Zhu

**Affiliations:** 1 Yangtze River Fisheries Research Institute, Chinese Academy of Fishery Sciences, Wuhan 430223, China; caoyongqing@yfi.ac.cn (Y.C.); dw1286329002@163.com (N.W.); 2 State Key Laboratory of Mariculture Biobreeding and Sustainable Goods, Yellow Sea Fisheries Research Institute, Chinese Academy of Fishery Sciences, Qingdao 266071, China

**Keywords:** schizothoracinae, Qinghai–Tibet plateau, drainage evolution, polyploidy, rediploidization, population genomics, management units/evolutionarily significant unit (MU/ESU), conservation management

## Abstract

The Qinghai–Tibet Plateau and surrounding mountain regions form one of the world’s most distinctive freshwater environmental gradients. Schizothoracinae are among the most representative endemic fish lineages in these systems and provide a useful model for studying how drainage history, genome evolution, adaptation, and conservation interact. In this review, we synthesize schizothoracine research within an environment–evolution–conservation framework. We examine how drainage history and connectivity shape divergence and gene exchange, how polyploidy and genome remodeling provide the genomic background for adaptive inference, and how phenotypic and population-genomic evidence can be translated into conservation units and management priorities. Across current studies, cold-associated metabolic remodeling and UV-related DNA damage response and repair emerge as the most recurrent molecular themes, whereas hypoxia-related signals are more context-dependent. We further show that morphology, otolith chemistry, age–growth traits, and population structure can strengthen MU/ESU interpretation when integrated with genomic evidence. Future progress will depend on broader chromosome-level genome coverage, more systematic comparison of structural genomic variation, standardized stressor-linked designs, and denser sampling in geomorphic transition zones and putative hybrid regions.

## Simple Summary

Schizothoracinae are one of the most representative fish groups in plateau river networks and provide a useful model for studying how river history, environmental stress, and genome evolution interact. In this review, we connect evidence from drainage history, genomics, phenotype, and population structure within a single framework. We further show how these data can support conservation-unit delimitation and management priorities across basins.

## 1. Introduction

The Qinghai–Tibet Plateau and its surrounding mountain valleys form one of the world’s most distinctive freshwater environmental gradients. Across plateau rivers and lakes, aquatic organisms experience interacting pressures from low temperature, intense ultraviolet radiation, variable dissolved oxygen, strong hydrodynamics, and, in some systems, salinization/alkalinization and shifts in ionic composition [1,2,3,4]. These conditions make plateau waters a natural setting for examining how multiple environmental stressors interact with spatially structured river networks.

In such plateau–montane freshwater environments, the subfamily Schizothoracinae is among the most representative endemic fish lineages. It is distributed mainly across the Qinghai–Tibet Plateau and adjacent river–lake systems [5], and has generated substantial species and lineage diversity in multiple drainages of Sichuan [6], Yunnan [7], and Tibet [1]. This group shows not only a broad morphological gradient from relatively primitive to highly specialized forms [8,9], but also pervasive polyploid genomic backgrounds, making it a particularly informative system for studying the coupling among environmental pressures, genome evolution, and phenotypic adaptation [10]. Episodic uplift of the Qinghai–Tibet Plateau, Quaternary glacial–interglacial fluctuations, and repeated drainage reorganizations further provide a historical framework for lineage divergence and geographic patterning in these fishes [11,12]. At the same time, chromosome-level genomes and multi-omics data now make it increasingly possible to evaluate post-WGD structural remodeling, early rediploidization, and recurrent adaptive themes in a lineage- and region-aware manner [1,13,14,15,16]. However, these research directions are still often treated separately: taxonomy and phylogeny, geological history, polyploid genomics, adaptive candidates, phenotypic evidence, and conservation planning are commonly discussed as parallel topics rather than as parts of one evidence chain. This fragmentation makes it difficult to assess which conclusions are strongly supported across data types, which remain context-dependent, and how far current evidence can be translated into defensible management decisions.

**Figure 1:**
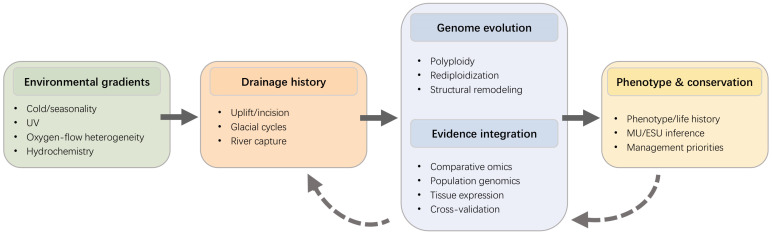
Conceptual evidence chain linking environment, genome evolution, phenotype, and conservation in schizothoracine fishes. In plateau river networks, environmental gradients and drainage history shape genome evolution, including polyploidy, structural remodeling, and early rediploidization. These processes are evaluated through integrated evidence from comparative omics, population genomics, tissue expression, and cross-validation, and are then linked to phenotypic and life-history variation, MU/ESU inference, and management priorities. Transition and contact zones require particular attention because introgression and secondary contact may decouple mitochondrial patterns from genome-wide structure and complicate MU/ESU delimitation. UV = ultraviolet radiation; MU = management unit; ESU = evolutionarily significant unit.

In this review, we synthesize schizothoracine research into an integrated environment–evolution–conservation framework across plateau river networks. Rather than treating historical drivers, genomic mechanisms, phenotypic outcomes, and conservation applications as separate themes, we connect them within a single evidence chain and use this structure to identify recurrent patterns, clarify current evidence limits, and translate population structure into operational MU/ESU hypotheses and conservation priorities (Figure 1).

## 2. Taxonomy and Phylogenetic Framework: Species Delimitation, Lineage Structure, and Geographic Patterns

### 2.1. Traditional Morphological Classification and Its Controversies

Morphology has long provided the foundation for schizothoracine taxonomy, especially through body form, mouth position, horny sheath development, barbel condition, and related trophic characters [17,18]. However, this morphology-based approach faces two systematic challenges. First, convergent and parallel adaptation is widespread in plateau river–lake environments, and many traits associated with hydrodynamics, substrate use, and feeding ecology recur across different lineages, so morphological similarity does not necessarily imply close relatedness [9,19,20]. Second, cryptic diversity and hybridization can further obscure species boundaries, as illustrated in the middle Yarlung Tsangpo, where *Schizothorax o’connori* and *S. waltoni* can remain morphologically recognizable while still showing evidence of natural hybridization [21]. Morphology therefore remains valuable for diagnosis and preliminary localization, but it should not be treated as a standalone basis for delimitation where contact, introgression, or strong ecological plasticity are plausible.

### 2.2. Molecular Phylogenetics and DNA Barcoding

Mitochondrial markers, including complete mitogenomes, remain useful in schizothoracines for rapid phylogeographic screening because they can efficiently reveal broad lineage structure, preliminary geographic partitioning, and cases of deep mitochondrial divergence. However, mtDNA represents a single, maternally inherited linkage unit and is therefore sensitive to introgression, mitochondrial replacement, and incomplete lineage sorting (ILS), especially under rapid radiation or persistent gene flow [22,23,24]. In addition, hybridization interacting with selection against incompatible combinations can maintain or even amplify mito–nuclear discordance, so mitochondrial patterns may fail to track genome-wide ancestry in contact zones [25]. Accordingly, mtDNA is best treated as a hypothesis-generation tool in schizothoracines, whereas robust species delimitation, species-tree inference, and evaluation of hybridization/introgression require nuclear genomic evidence and coalescent-aware models that explicitly test gene flow versus ILS [7,26,27,28]. Treating mtDNA as preliminary evidence and nuclear data as decision-grade validation helps avoid ambiguous statements and improves cross-basin comparability [24,28].

### 2.3. From Tree Building to Process Inference

Multilocus and genome-wide datasets have shifted schizothoracine systematics from describing patterns to testing processes. By integrating nuclear markers, population-genomic structure, and model-based inference, current studies are better able to distinguish incomplete lineage sorting from introgression and to evaluate whether observed lineage structure reflects persistent divergence, secondary contact, or both [27,29]. This transition is especially important in plateau river networks, where drainage history, hybridization, and heterogeneous connectivity can all leave overlapping signals in phylogenetic and population data. In this sense, the key advance is not simply the construction of better trees, but the ability to explain them in a historically and geographically explicit framework.

### 2.4. Natural Hybridization and Species Boundaries

Species boundaries in schizothoracines are often better understood as dynamic segments within a divergence–secondary contact–hybridization continuum rather than as fixed lines. A representative example comes from the middle Yarlung Tsangpo River, where natural hybridization between *Schizothorax o’connori* and *S. waltoni* demonstrates that lineage contact can persist even where parental forms remain recognizable [21]. This case is important not simply because hybrids occur, but because it shows that contact zones can become the decisive settings for testing whether observed variation reflects stable divergence, ongoing introgression, or both.

Accordingly, when hybrid risk is high or divergence is shallow, morphology or mtDNA alone cannot provide decision-grade delimitation. In such reaches, nuclear genomic data are required to evaluate the extent and direction of introgression, to assess genome-wide reproductive isolation, and to support taxonomic revision and operational MU/ESU delineation in a defensible way [26,27].

## 3. Geologic History and Drainage Evolution as Drivers of Radiation: From “Plateau Uplift” to “Lineage Divergence”

### 3.1. Geological Processes, Drainage Rearrangement, and Genetic Divergence

Geological uplift, glacial–interglacial dynamics, and drainage rearrangement have jointly shaped the evolutionary setting of plateau river networks by repeatedly altering connectivity among reaches and basins. Rather than producing a single uniform outcome, these processes create alternating conditions of fragmentation, reconnection, and secondary contact, thereby generating structured expectations for lineage divergence and gene exchange [11,28,30]. In this context, canyon incision, river capture, and glacially mediated hydrological shifts are best interpreted not simply as historical background, but as mechanisms that help explain why some lineages remain isolated whereas others retain signals of admixture or basin–boundary discordance [28,30,31].

### 3.2. The Yarlung Tsangpo as a Representative Drainage Case

The Yarlung Tsangpo River system provides one of the most informative plateau examples for linking drainage history to lineage divergence because it combines strong geomorphic structuring with opportunities for secondary contact. Evidence from this system indicates that population differentiation and local gene exchange can coexist within the same drainage, rather than forming a simple pattern of complete isolation among lineages [21,32].

The broader significance of this case is that historically dynamic plateau rivers may contain both divergence zones and contact zones within a single network, so population history cannot be inferred reliably from basin assignment alone. In such systems, genome-wide and population-scale data are especially important for distinguishing persistent divergence from ongoing introgression and for interpreting lineage structure in a way that is useful for later conservation-unit delimitation [32].

### 3.3. A Representative Case: The Tianshan–Pamir Corridor, Westward Dispersal, and Rapid Diversification

The Tianshan–Pamir region provides a useful contrast to plateau-core drainage systems because lineage diversification there can also be interpreted within a drainage-corridor framework rather than by present basin labels alone. Existing evidence suggests that historical dispersal and subsequent differentiation may have proceeded westward across interconnected river systems, but where gene flow remains plausible, dispersal direction and introgression must still be tested with nuclear genomic and population-scale data [33].

Drainage history in plateau river networks is therefore best treated as a predictive framework for divergence, secondary contact, and basin–boundary discordance rather than as background context alone. This is why transition zones and tributary junctions are especially informative for later genome-based interpretation and for delimiting biologically meaningful conservation units.

## 4. Polyploidy and Genome Evolution: From Ploidy Patterns to Early Rediploidization

### 4.1. Ploidy Landscape and Core Questions

Against this drainage-history background, schizothoracines show marked ploidy complexity, with tetraploid and even higher-ploidy lineages reported across multiple drainage systems [28,34,35]. The goal of this chapter is therefore not to re-establish the occurrence of polyploidization, but to examine how the timing and mode of genome doubling may differ among lineages and how duplicated genomes move toward long-term stability through rediploidization and structural remodeling. Because direct evidence is still concentrated in a limited number of high-quality genomes and population datasets, many mechanistic interpretations remain provisional and require broader lineage sampling for validation.

### 4.2. “Young Tetraploids” and Early Rediploidization

Within current studies of schizothoracine polyploidy, the tetraploid reference genome of *Schizothorax o’connori* serves as a key anchor. Using a single analytical framework, it provides a time-scale estimate for whole-genome duplication (WGD) and links post-duplication remodeling to early rediploidization, thereby offering a traceable chain from genome doubling to genome restructuring [10,36]. Chromosome-scale assemblies further make this process structurally testable by identifying homeologous chromosomes and syntenic blocks expected under tetraploidy [37,38].

Importantly, “young tetraploid” does not imply the absence of rediploidization. Some genomic regions may show earlier restriction of recombination, accumulation of structural differences, or regulatory divergence, including tissue- or condition-specific expression shifts [39,40]. In *S. o’connori*, the main value of chromosome-scale evidence is therefore not simply to confirm polyploidy, but to show that early rediploidization can already be tracked through structural correspondence, regionally uneven divergence, and regulatory partitioning.

### 4.3. Evidence Framework for Polyploid Origins and Post-WGD Processes

Debates over the origin of polyploidy in schizothoracines usually focus on autopolyploidization versus allopolyploidization [41]. However, this distinction is best treated as an integrative diagnosis rather than as a binary label derived from any single signal, because incomplete lineage sorting (ILS), incomplete sampling, and river-network–mediated secondary contact can blur otherwise diagnostic patterns [7,28,42].

Homeolog phylogenetic placement can provide directional clues—for example, preferential clustering of homeologs with different progenitor lineages is more compatible with allopolyploidy—but gene-tree patterns may be distorted where admixture is plausible. In such cases, genome-wide tests that explicitly evaluate reticulation or admixture are needed to complement gene-tree evidence [13,25,43,44,45]. Similarly, subgenome asymmetry in divergence, TE landscapes, and retention or expression is often discussed as support for allopolyploidy, whereas early symmetry may be more compatible with autopolyploidy; however, these patterns can be reshaped by rediploidization, homeologous exchange, and lineage-specific TE activity, and should therefore be treated as supportive rather than decisive [13,36,43,44,46].

Because pairing behavior and inheritance mode differ between auto- and allopolyploids, cytogenetic and segregation evidence provides an important constraint that sequence-based arguments often lack, but such data remain scarce across schizothoracine lineages [42]. Chromosome-level assemblies therefore offer the most structurally testable anchor by resolving homeologous chromosome correspondence and duplicated blocks, allowing origin hypotheses, rediploidization, demographic history, and gene flow to be assessed within the same coordinate framework [10,36,37,38,46]. Given documented hybridization and gene flow in plateau rivers [32] and explicit ILS-versus-gene-flow analyses in schizothoracines [7,28], future work should prioritize chromosome-scale homeolog correspondence together with genome-wide reticulation tests in sampling designs that target geomorphic transition zones and putative contact regions [10,21,25,28,42,45].

### 4.4. Comparison and Synthesis

Studies of other polyploid cyprinids suggest that rediploidization often proceeds in a regionally heterogeneous, mosaic manner: some chromosomal segments are more prone to structural rearrangement or homeologous exchange, TE landscapes can help trace separation histories, and duplicated genes frequently show biased retention or expression divergence [38,46]. In schizothoracines, this process is especially important to interpret because complex ploidy histories are embedded within strong environmental gradients and dynamic drainage reorganization. A key unresolved question is therefore whether the spatiotemporal trajectory of rediploidization has also been shaped by geological history, basin connectivity, and long-term environmental heterogeneity.

From this perspective, genomic structural remodeling is best viewed as a testable substrate for later adaptive inference rather than as an endpoint in itself. Structural variation, TE-associated insertions, and region-specific rediploidization may alter gene dosage and regulatory context, thereby affecting tissue- and condition-dependent expression of duplicated stress-response modules [39,40]. However, this possibility still requires broader comparative evaluation across lineages. The most important next step is therefore to obtain chromosome-level genomes and comparable population datasets across multiple schizothoracine lineages, so that structural heterogeneity can be examined in the same coordinate framework as population divergence and gene-flow signals [10,36,37,38,46]. How such genomic remodeling maps onto specific environmental stress-response patterns is considered in Section 5 and Section 6.

## 5. Omics Resources and Methodological Advances: From Data Accumulation to Testable Evidence Chains

### 5.1. From Single Genomes to Comparative Resource Landscapes

Genomic resources for schizothoracines have expanded from isolated reference genomes to a broader landscape that now includes chromosome-scale assemblies, whole-genome resequencing, transcriptomes, and comparative datasets across multiple plateau fishes [10,47,48,49]. These resources have made it possible to move beyond simple species description and begin testing questions about genome duplication, structural remodeling, divergence, and adaptive inference in a more explicit comparative framework.

A persistent limitation, however, is the uneven distribution of available data. High-quality genomes and dense population sampling remain concentrated in a small number of representative species and basins, whereas several key lineages, geomorphic transition zones, and putative contact regions are still under-sampled [10,47,48]. In this sense, the main challenge is no longer simple data scarcity, but evidentiary imbalance: some systems are becoming rich enough for integrative inference, while others still lack the genomic and population coverage needed for reliable cross-basin comparison.

### 5.2. Key Analytical Pipelines

Current schizothoracine omics studies are most useful when analytical workflows are viewed in terms of the questions they can answer, rather than as isolated technical procedures. At the genome scale, chromosome-level assemblies provide the strongest structural anchor for testing polyploidy-related questions, because they allow homeologous correspondence, duplicated blocks, and regionally heterogeneous rediploidization to be evaluated within a shared coordinate framework [10,47,48,49]. In this sense, the main value of genome-scale analysis is not procedural complexity itself, but the ability to connect structure, duplication history, and later population-genomic interpretation in one reference system.

At the comparative level, cross-species genomic and transcriptomic datasets are most informative for identifying recurrent functional modules rather than single “adaptive genes.” By combining ortholog-based comparisons, gene-family or sequence-evolution analyses, and tissue-level expression evidence, existing studies can repeatedly recover candidate themes related to metabolism, DNA repair, ion transport, and other stress-relevant functions [50,51]. However, the reusability of these conclusions depends strongly on data balance across species, tissues, and lineages; broader and more comparable datasets are therefore more important than adding further method-specific complexity.

At the population scale, the most powerful workflows are those that connect geographic structure, historical processes, and candidate adaptive signals within the same evidence chain. In schizothoracines, whole-genome resequencing and related population-genomic datasets already provide direct nuclear-genomic entry points for interpreting basin divergence, demographic history, and candidate loci, and for later translation to connectivity assessment and MU/ESU hypotheses [7,14]. Transcriptomic analyses are most valuable when they return these candidate signals to physiological context by testing whether inferred modules are expressed in relevant tissues or stress conditions [10,14,52]. Overall, the main limitation is no longer the absence of analytical routes, but the uneven distribution of evidence across lineages, basins, and transition zones. Accordingly, the most urgent next steps are broader structural comparisons, more standardized stressor-linked designs, and denser sampling in geomorphic transition zones and putative hybrid regions.

## 6. Molecular Mechanisms of High-Altitude Adaptation: Shared Themes and What Makes “Aquatic High-Altitude Adaptation” Distinct

**Figure 2:**
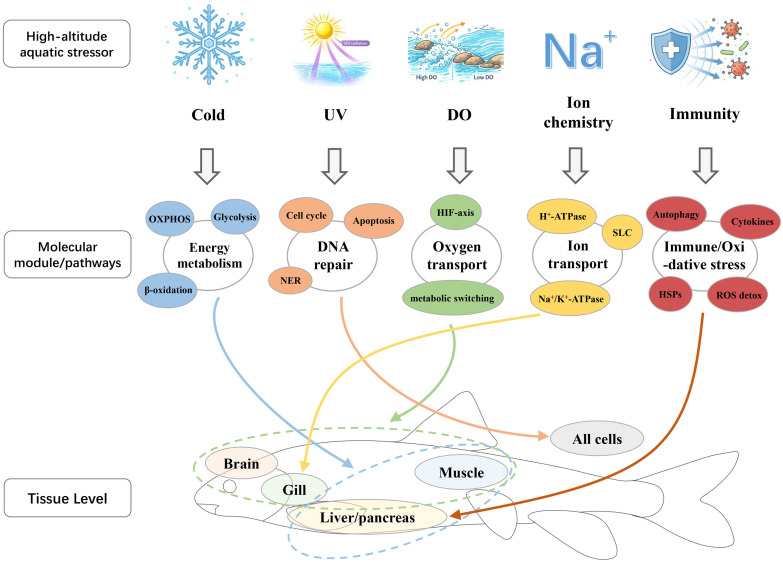
Mechanistic inference framework linking major high-altitude stressors, candidate molecular modules, and key tissues in schizothoracine fishes. The figure summarizes representative pathways and tissue targets commonly examined in studies of cold, UV, dissolved oxygen heterogeneity, ion chemistry, and immune or oxidative stress responses. Dashed outlines indicate grouped tissue targets used when a single module is linked to multiple tissues. The figure is intended as a guide to mechanistic interpretation and stress-response profiling rather than as evidence that all stressors show equal strength or consistency across datasets. UV = ultraviolet radiation; DO = dissolved oxygen; OXPHOS = oxidative phosphorylation; NER = nucleotide excision repair; SLC = solute carrier family; HSPs = heat shock proteins; ROS = reactive oxygen species.

This chapter organizes molecular evidence for high-altitude adaptation in schizothoracines by major stressors, prioritizing topics for which existing data can support a closed loop of “comparative omics–population divergence–tissue expression/stress responses.” Key tissues frequently used for mechanistic inference and stress-response profiling are summarized in Figure 2. For modules where evidence is fragmented or debated, we specify the scope of applicability and the key steps that remain to be tested.

### 6.1. Cold Stress and Metabolic Remodeling

High-altitude lakes and rivers typically experience long ice-covered periods and short ice-free seasons, which strongly constrain productivity and compress biological activity into a brief seasonal window [49,53]. Under these conditions, cold adaptation in schizothoracines is better interpreted as system-level remodeling of energy balance than as variation in a small set of “cold genes”. Comparative genomics, population-genomic scans, and transcriptomic studies repeatedly converge on pathways related to energy metabolism, especially OXPHOS and central carbon/lipid metabolism [10,54]. Across current datasets, the strongest support comes when genomic candidates and tissue-level expression patterns point to the same metabolic modules under cold or closely related stress contexts [15,55].

### 6.2. Intense UV Radiation and DNA Damage Repair

UV exposure in high-altitude waters is shaped by water clarity, suspended material, and depth use, but in clear plateau lakes and river reaches it can penetrate deeply enough to impose persistent DNA damage and genome-maintenance pressure [56]. In schizothoracines and related high-altitude cyprinids, comparative genomics and population resequencing repeatedly recover DNA damage response pathways, especially NER-related functions, as a relatively stable candidate axis of adaptation [10,14]. Transcriptomic studies further support this interpretation by repeatedly detecting tissue-dependent regulation of oxidative-damage responses, cell-cycle control, apoptosis, and repair-associated networks under stress exposure [53,57].

### 6.3. Hypoxia: Inconsistent Signals

Compared with cold- and UV-related modules, hypoxia-associated genomic signals in schizothoracines are often less recurrent and less consistently recovered in broad selection or enrichment analyses. However, this should not be interpreted as evidence that hypoxia adaptation is absent. Instead, targeted comparative and functional evidence, especially for the HIF-α family, suggests that adaptive change is present but may be more difficult to detect as a strong, uniform sequence-level signature across studies [58].

A likely reason is that hypoxia in plateau rivers is rarely a simple, chronic, and spatially uniform exposure. Dissolved oxygen can vary sharply across reaches, seasons, and flow conditions, while turbulence and hydrodynamic reaeration may rapidly modify local oxygen regimes [59,60]. In addition, behavioral selection of flow conditions and microhabitats can buffer effective hypoxia exposure, further weakening the expectation of repeated “one-pathway” genomic convergence [61]. Under such conditions, adaptation to oxygen variability may proceed through multiple routes, including classical hypoxia regulation, shifts in oxygen uptake or transport capacity, metabolic adjustment, and reversible plastic responses during hypoxia–reoxygenation cycles [62,63].

Taken together, current evidence suggests that hypoxia is better treated as a context-dependent and exposure-regime-sensitive stressor than as a uniformly recurrent genomic module. Future studies should therefore combine genomic or transcriptomic sampling with better-resolved environmental characterization, including reach- and time-specific DO profiling, so that differences among studies can be interpreted against quantified oxygen regimes rather than against an overly broad category of “hypoxia”.

### 6.4. Osmoregulation, Ion Homeostasis, and Water Chemistry

Plateau lakes and rivers show pronounced heterogeneity in salinity, pH, hardness, and major-ion composition, and many plateau lakes are distinctly saline–alkaline [64]. Accordingly, water-chemistry-associated adaptation is most likely to concentrate in functional modules related to ion transport, osmoregulation, and acid–base homeostasis. Comparative genomic and multi-omics studies in schizothoracines and related plateau cyprinids repeatedly detect accelerated evolution and/or differential expression in such genes, supporting the view that this can be treated as a water-specific divergence module and retained as a priority target for cross-system comparison under contrasting hydrochemical regimes [1,15,65]. Future progress will depend less on adding more isolated candidate lists than on linking population-genomic structure to quantified hydrochemical gradients in a comparable framework across basins.

### 6.5. Immunity and Stress Responses

Immune- and stress-related processes are frequently implicated in schizothoracine studies and often recur in comparative or phylogenetic analyses as part of broader high-altitude candidate themes [51]. Tissue transcriptomics and stress-exposure experiments further show coordinated shifts in immune genes together with HSPs, oxidative-stress/ROS responses, and cell-cycle or apoptosis-related networks [57]. These signals vary strongly with tissue type, stress severity, and sampling window, so they are best interpreted as components of a coupled immune–metabolic–oxidative stress response rather than as isolated pathway readouts. A cautious synthesis is therefore that schizothoracines often exhibit integrated stress-response networks under multiple stress contexts, whereas the relative roles of long-term genetic adaptation and short-term plasticity still require standardized exposure designs linked to phenotype and life-history evidence in the next chapter.

Across the stressors reviewed here, cold-associated metabolic remodeling and UV-related genome maintenance emerge as the most recurrent molecular themes across current datasets. By contrast, hypoxia-related signals appear more context-dependent, likely reflecting heterogeneous exposure regimes, multiple physiological routes, and a stronger contribution of plasticity. Water-chemistry-associated divergence and integrated immune–oxidative stress responses occupy an intermediate position: repeatedly implicated, but still more sensitive to basin context, tissue choice, and exposure design. This hierarchy of evidence strength should guide both future validation and the caution with which adaptive candidates are translated into broader ecological or management conclusions.

## 7. Phenotypic, Morphological, and Life-History Adaptation: Linking “Molecular Modules” to Ecological Function

This chapter synthesizes testable evidence at the phenotypic and life-history levels. It highlights reusable evidence chains in morphometrics, otolith-based approaches, and age–growth analyses in schizothoracines, providing support for subsequent MU/ESU delineation and the pressure–management sections.

### 7.1. Morphological Differentiation and Niche Association

Morphological variation remains an important entry point for taxonomic identification and ecological interpretation in schizothoracines, especially in plateau river–lake systems where flow regimes and food-resource gradients can shape body form, fin morphology, and feeding-related structures [18]. However, under convergence, plasticity, and hybridization/introgression, morphology is most useful for indication and localization rather than as a standalone basis for definitive inference [9,22]. Current work therefore combines two main approaches: geometric or multivariate morphometrics to improve discrimination among closely related or geographically structured groups [66,67], and explicit functional interpretation of body, fin, and feeding-related traits in relation to habitat gradients such as flow, substrate, and food resources [9,68]. Because morphology is also influenced by ontogeny, reproductive condition, temperature, flow, and food availability, within-species variation can be large enough to confound morphology-only assignment across habitats or sampling periods [69,70].

### 7.2. Otolith Microchemistry and Life-History Reconstruction

When integrated with annulus information, core-to-edge otolith chemical transects can be mapped onto ontogenetic stages, allowing reconstruction of movement timing and residency across an individual’s lifetime [71,72]. In schizothoracines, within-basin differences in spawning-ground water chemistry may also be reflected in elemental ratios along the growth axis, enabling inference of early origin and key life-history transitions [73]. Under fragmentation or damming, otolith microchemistry is especially useful for evaluating contemporary movement across reaches, whereas population genetic structure more directly reflects intergenerational gene flow and longer-term isolation [74,75]. Because elemental incorporation depends on spatially and temporally varying baselines, otolith-based inference should be anchored in characterized multi-element baselines with replicate sampling [76].

### 7.3. Age and Growth

Across many schizothoracine lineages, low temperature and a short growing season are associated with a recurring life-history syndrome of slow growth, longevity, and late maturation. These traits are not only functional endpoints of high-elevation adaptation, but also parameters of direct importance for fisheries assessment and management. In *Schizothorax o’connori*, otolith-based studies indicate long lifespan and low von Bertalanffy growth coefficients, consistent with a classic long-lived, slow-growing strategy [77,78]. From a management perspective, similar life-history profiles in *Oxygymnocypris stewartii* and *S. waltoni* imply elevated sensitivity to overfishing and other disturbances, especially because delayed maturation and longevity increase the importance of protecting older reproductive individuals [11,79]. At the same time, age-and-growth inference remains reliable only when annulus formation is seasonally validated and age readings are quality controlled, particularly in older fish where otolith-based estimates may diverge [78,80].

### 7.4. Reproductive Ecology and Trophic Ecology

Although evidence remains uneven across species and basins, reproductive ecology provides some of the most directly actionable information for schizothoracine conservation. Data on spawning grounds, spawning microhabitats, and the timing of reproduction relative to water temperature and discharge can directly inform reserve placement, ecological-flow management, and habitat restoration [81]. Recent work using acoustic telemetry and hydrodynamic–substrate surveys has further helped define key reaches and key seasonal windows for management action [16].

By contrast, trophic ecology currently serves mainly as a supplementary indicator stream. Gut microbiome and related physiological evidence can reflect diet- and habitat-associated differences among schizothoracine fishes [82,83,84,85], but these signals are too sensitive to season and local environmental context to function as a primary basis for delimitation or management decisions.

Taken together, the phenotype streams reviewed here are not equally informative for conservation translation. Morphology and high-throughput phenotyping are most useful for screening, otolith microchemistry for movement and reach use, and age–growth plus reproductive ecology for vulnerability and management timing. Trophic ecology is better treated as a supplementary indicator, and the main value of Section 7 is therefore to strengthen MU/ESU interpretation when combined with genomic structure.

## 8. Population Genetic Structure and Conservation Units: From Genetic Diversity to Management Units (MU/ESU)

Population genetic structure in schizothoracines is strongly shaped by river-network spatial organization, including reach fragmentation, tributary separation, and barrier effects. This chapter synthesizes how such spatial differentiation can be translated into operational MU/ESU hypotheses and management-relevant conservation decisions.

### 8.1. Operationalizing MU/ESU in Schizothoracine Research

In schizothoracines, a management unit (MU) is generally used for population units at contemporary or near-contemporary timescales where gene flow is too limited to maintain demographic connectivity, whereas an evolutionarily significant unit (ESU) emphasizes longer-term evolutionary independence [86,87,88]. In plateau river networks, however, these categories cannot be operationalized by fixed thresholds alone, because dendritic topology, asymmetric dispersal, barriers, and contact zones can all shape population structure in ways that blur simple boundary assignment [89]. MU/ESU delimitation should therefore depend on evidence-chain completeness, including sampling of transition zones, evaluation of barrier versus distance effects, and explicit consideration of admixture or hybridization [86,87]. Under these conditions, the most defensible unit hypotheses are those that connect nuclear-genomic structure to management-relevant ecological and life-history context.

### 8.2. Upgrading Data Types and Elevating Evidence Strength

In schizothoracines, MU/ESU inference has moved from limited marker sets to genome-wide evidence [28]. However, the value of such inference depends strongly on how well species boundaries are resolved, because cryptic diversity and introgression can decouple mtDNA patterns from genome-wide structure and cause limited marker panels to over- or under-estimate unit boundaries [21,22,24]. Genome-wide SNP datasets and WGS resequencing now provide stronger support by allowing explicit tests of admixture, introgression directionality, and concordance between geographic structure and nuclear variation [8,90]. This upgrade is especially important where enhancement stocking, translocation, or aquaculture-related introductions may erode local unit integrity, making traceable broodstock sourcing and MU-aligned management more necessary [91,92,93,94]. Recent GBS and WGS studies in schizothoracines, including work on *Schizothorax kozlovi*, show that conservation-unit inference can now move beyond sparse-marker clustering toward integrated evaluation of divergence, demographic risk, and management relevance [8,95].

### 8.3. Multi-Scale Evidence Chains and Management Translation

*Schizothorax o’connori* in the Yarlung Tsangpo system is among the schizothoracine taxa for which a relatively complete conservation–genetic evidence loop can be assembled. A chromosome-level reference genome provides a unified framework for variant discovery and population comparison, and WGS resequencing further links population structure, connectivity differences, genetic diversity and risk, and candidate adaptive signals within the same inferential framework [10].

In this system, MU delineation is strongest when sampling covers main reaches, major tributaries, and geomorphic transition or contact zones, and when genome-wide data are used to evaluate structure, differentiation, admixture, and demographic history together rather than as separate signals [7,10,28,86,87,93]. This is particularly important because mtDNA or sparse marker panels may be decoupled from nuclear structure under cryptic diversity and secondary contact [21,22,24]. Where divergence aligns consistently with geographic units and is accompanied by low diversity or signals consistent with bottlenecks, a precautionary management implication is to minimize translocation and stocking across units while prioritizing monitoring in transition zones [10,93].

At the population-genomic level, current WGS studies in *S. o’connori* allow divergence patterns to be interpreted in relation to canyon geomorphology, drainage connectivity, and potential barrier effects, while also informing conservation prioritization, broodstock sourcing, and risk control for cross-reach translocation [14,94,96]. Based on currently available evidence, MU delineation in this species can be hypothesized to follow major reaches and tributary systems, with intensified sampling in transition zones and putative contact areas to test boundary stability. Coupling such measures with long-term monitoring of connectivity and key habitat use allows MU boundaries to be updated dynamically as new evidence accumulates [14,96].

Important gaps remain in under-sampled transition zones and putative introgression areas, where nuclear-genomic validation is still limited. Accordingly, MU/ESU hypotheses in these systems should remain provisional, with monitoring prioritized and stocking or translocation decisions kept conservative until unit boundaries are better resolved.

## 9. Anthropogenic Pressures and Management Responses: Translating Evidence into an Actionable Checklist

With MU/ESU hypotheses in place (Section 8), schizothoracines across plateau drainages often combine life-history vulnerability with increasing human pressures. The most commonly reported risks include hydropower-related fragmentation and altered flow–temperature regimes, degradation of key spawning and nursery reaches, and fishing pressure, all of which can reduce connectivity, shrink functional habitats, and increase genetic and demographic risk [11,90,97,98,99,100,101,102]. The section below therefore summarizes management responses around three major pressure types at the “key reach–key seasonal window” scale.

Because the relative importance of these drivers differs among species and basins, the framework below is intended as a minimum transferable set of actions rather than a full diagnosis for each taxon. In practice, implementation should still be adjusted to local MU boundaries, focal reaches, and seasonal windows.

### 9.1. Minimum Actionable Response Framework for Three Major Pressure Types

Because many schizothoracine taxa exhibit slow growth, late maturation, and long lifespans, recovery is often more sensitive to size/age structure and recruitment bottlenecks than to short-term fluctuations in total catch [11,97]. Harvest regulation should therefore focus not only on total yield, but also on protecting population structure through minimum legal size and seasonal closures during spawning. In severely depleted reaches, additional restrictions on large-bodied individuals may further reduce dependence on a small number of breeders [98].

Under engineering disturbance and reach fragmentation, management should set explicit connectivity targets during key migration periods and evaluate them using a small number of repeatable indicators, such as arrival efficiency, passage efficiency, and the number of passable days under standardized discharge conditions [90,99]. Enhancement stocking and cross-reach translocations should also remain aligned with MU boundaries. Where genetic clustering corresponds closely to geographic units, a conservative default is “source within the same MU and release within the same MU,” combined with intensified monitoring on both sides of transition zones or suspected barriers to reduce the risk of genetic swamping or outbreeding and to support later updates of MU delineation [103].

Habitat degradation and altered hydrological regimes commonly weaken juvenile recruitment through two linked pathways: mismatch in the flow–temperature regime that supports spawning and early development, and shrinkage of suitable spawning and nursery microhabitats [100,101,104]. Restoration and ecological-flow management are therefore most effective when organized around a “key reach–key time window–key microhabitat” unit. In practice, this means prioritizing spawning grounds, nursery shallows, and overwintering pools as functional units, identifying critical seasonal windows for reproduction and early development, and guiding ecological regulation and habitat restoration toward the periods and locations that contribute most to recruitment [101,102,105].

### 9.2. One-Page Action Checklist and Evaluation Metrics

At the implementation level, management actions can be condensed into a tiered “one-page” checklist: enforce a closed season and minimum legal size, establish fixed monitoring stations in transition zones, match stocking sources to MUs by default, complete functional zoning of key reaches, and quantify passage or habitat performance during critical seasonal windows [81,91,106]. To keep implementation manageable, evaluation can be limited to three indicator groups: connectivity (e.g., arrival/passage efficiency or passable days), population status (juvenile recruitment and body-length/age structure), and management risk (stocking-source compliance and early-warning signals in transition zones). If these core indicators fail to improve over time, management should adjust flow regulation, relocate restoration interventions, or tighten harvest and stocking rules [105].

The indicators summarized in Table 1 are consistent with recent connectivity and management-effectiveness studies that emphasize objective-based performance monitoring and adaptive adjustment [90,99,107]. In this sense, Table 1 is best viewed as an iterative loop: quantify performance during key windows, evaluate whether core indicators improve, and modify engineering, flow, habitat, or harvest measures when they do not [105]. At the same time, where populations are severely depleted or in-river recovery is slow, ex situ safeguards such as germplasm cryobanking or assurance broodstocks may serve as cautious complements to habitat and connectivity restoration [108].

Overall, MU boundaries, critical seasonal windows, and quantified connectivity and habitat metrics can provide an integrating framework that places genetic structure, life-history vulnerability, and engineering interventions into a common management context.

## 10. Integrated Framework and Outlook

Research on schizothoracine fishes can be condensed into a transferable evidence chain: river-network history and environmental gradients shape divergence and selection; against a background of polyploidy and genome remodeling, recurrent molecular modules emerge; these are then expressed through phenotype, life history, and ecological function, and finally translated into conservation units and management responses. The next priority is therefore not to expand a checklist of phenomena, but to make this chain more verifiable and comparable across species and basins.

Looking ahead, debated issues should be reformulated as testable questions. Progress will depend less on accumulating more isolated signals than on identifying recurrent modules, explaining why they recur, and linking divergence, phenotype, and ecological process within the same spatial and temporal framework. At the same time, scientific outputs should be compressed into a smaller set of shared indicators that are easier to compare across basins and studies.

Future work should therefore prioritize transition-zone-aware sampling, reproducible metadata standards, and a core set of cross-study comparable indicators. With these foundations, schizothoracine research can move more reliably from case-driven findings to framework-driven synthesis and provide more consistent support for conservation-unit inference and biodiversity conservation in plateau river networks.

## 11. Conclusions

Schizothoracinae in plateau river networks provide a useful model for linking environmental gradients, genome evolution, and conservation. Across current studies, cold- and UV-related responses emerge as the most recurrent molecular themes, whereas hypoxia-related signals are more context-dependent.

By integrating genomic, phenotypic, and population-structure evidence, current research can increasingly translate divergence patterns into operational MU/ESU hypotheses and management priorities. Future progress will depend on broader chromosome-level genome coverage, more systematic comparison of structural genomic variation, standardized stressor-linked designs, and denser sampling in geomorphic transition zones and putative hybrid regions.

## Figures and Tables

**Table 1:** Translating MU/ESU evidence into management actions and performance indicators.

Objective	Minimum Action	Indicator	Focus	Data	Adjustment	Reference
Structure protection	Closed season + Min size	Size/age rebound; recruitment ↑	Key reaches; spawning season	P	No improvement for 2 years → tighten	[11,97,98,105]
Connectivity	Set passage target	Arrival efficiency; passage efficiency; number of passable days	Migration season; representative reaches	C	No improvement for 2 years → tighten	[81,90,99,101,106,105]
Stocking risk control	MU-aligned source & release with ploidy/hybrid screening when needed	Source compliance; anomaly warning	Transition zone	G + R (add ploidy/hybrid diagnostics in contact zones)	Violation → stop/revise	[88,89,103]
Habitat restoration	Microhabitat restoration	Depth/velocity/substrate/cover area	Key reach	H	No improvement for 2 years → tighten	[100,101,102,105,109]

This table summarizes a minimum set of management actions and evaluation metrics for schizothoracine conservation, based on the evidence synthesized in Section 7, Section 8 and Section 9. Data codes: G, genetics/population genomics (SNP/GBS/WGS; MU delineation and risk assessment); P, population status (juvenile recruitment; size/age structure); C, connectivity monitoring (arrival/passage efficiency; passable days); H, habitat survey (depth, velocity, substrate, cover structures, and effective area); R, stocking registry (broodstock/juvenile origin records).

## Data Availability

No new data were created or analyzed in this study. Data sharing is not applicable to this article.

## References

[1] Tian F., Liu S., Zhou B., Tang Y., Zhang Y., Zhang C., Zhao K. (2022). Chromosome-level genome of Tibetan naked carp (*Gymnocypris przewalskii*) provides insights into Tibetan highland adaptation. DNA Res..

[2] Xie Y.G., Qi Y.L., Luo Z.H., Qu Y.N., Yang J., Liu S.Q., Yang H.L., Xie D.W., Wang Z., Jiang H.C. (2025). Lake salinization on the Qinghai-Tibetan Plateau alters viral community composition and lifestyles. Commun. Earth Environ..

[3] Ren W., Gao Y., Qian H., Qu W., Shi X., Ma Y., Su Z., Ma W. (2025). The Evolution and Drivers of Hydrochemistry in Nam Co Lake, the Third Largest Lake on the Tibetan Plateau, over the Last 20 Years. Sustainability.

[4] Grabner D., Rothe L.E., Sures B. (2023). Parasites and Pollutants: Effects of Multiple Stressors on Aquatic Organisms. Environ. Toxicol. Chem..

[5] Du T., Ding C., Yang K., Chen J., Liu X., Lv W., Ding L., He D., Tao J. (2024). A global dataset on species occurrences and functional traits of *Schizothoracinae* fish. Sci. Data.

[6] He Z., Gao K., Chen H., Yang D., Pu Y., Zheng L., Jiao Y., Xiong J., Chen Q., Lai B. (2023). Comparative Population Dy-namics of *Schizothorax wangchiachii* (*Cyprinidae*: *Schizothoracinae*) in the Middle Reaches of the Yalong River and the Upper Reaches of the Jinsha River, China. Animals.

[7] Chen W., Yue X., He S. (2017). Genetic differentiation of the *Schizothorax* species complex (*Cyprinidae*) in the Nujiang River (upper Salween). Sci. Rep..

[8] Zhou C., Xiao S., Liu Y., Mou Z., Zhou J., Pan Y., Zhang C., Wang J., Deng X., Zou M. (2020). Comprehensive transcrip-tome data for endemic *Schizothoracinae* fish in the Tibetan Plateau. Sci. Data.

[9] Tang Y., Li C., Wanghe K., Feng C., Tong C., Tian F., Zhao K. (2019). Convergent evolution misled taxonomy in schizothoracine fishes (*Cypriniformes*: *Cyprinidae*). Mol. Phylogenet. Evol..

[10] Xiao S., Mou Z., Fan D., Zhou H., Zou M., Zou Y., Zhou C., Yang R., Liu J., Zhu S. (2020). Genome of Tetraploid Fish *Schizothorax o’connori* Provides Insights into Early Re-diploidization and High-Altitude Adaptation. iScience.

[11] Guo X.Z., Zhang G.R., Wei K.J., Ji W., Yan R.J., Wei Q.W., Gardner J.P.A. (2019). Phylogeography of the threatened tetraploid fish, *Schizothorax waltoni*, in the Yarlung Tsangpo River on the southern Qinghai-Tibet Plateau: Implications for conservation. Sci. Rep..

[12] Regmi B., Douglas M.R., Wangchuk K., Zbinden Z.D., Edds D.R., Tshering S., Douglas M.E. (2023). The Himalayan uplift and evolution of aquatic biodiversity across Asia: Snowtrout (*Cyprininae*: *Schizothorax*) as a test case. PLoS ONE.

[13] Xu M.R., Liao Z.Y., Brock J.R., Du K., Li G.Y., Chen Z.Q., Wang Y.H., Gao Z.N., Agarwal G., Wei K.H. (2023). Maternal dominance contributes to subgenome differentiation in allopolyploid fishes. Nat. Commun..

[14] Gao K., He Z., Xiong J., Chen Q., Lai B., Liu F., Chen P., Chen M., Luo W., Huang J. (2024). Population structure and adaptability analysis of *Schizothorax o’connori* based on whole-genome resequencing. BMC Genom..

[15] Zhou B., Sui R., Yu L., Qi D., Fu S., Luo Y., Qi H., Li X., Zhao K., Liu S. (2025). Transcriptomics and proteomics pro-vide insights into the adaptative strategies of Tibetan naked carps (*Gymnocypris przewalskii*) to saline-alkaline variations. BMC Genom..

[16] Li B., Hu F., Li W., Su W., Zhu J., Jiang W. (2025). Spawning habitat selection in *Schizothorax wangchiachii* using acoustic tagging and tracking. Front. Ecol. Evol..

[17] Cao W.X., Chen Y.Y., Wu Y.F., Zhu S.Q., Tibetan Expedition Team of the Chinese Academy of Sciences (1981). Origin and evolution of *Schizothoracine* fishes in relation to the upheaval of the Xizang Plateau. Studies on the Period, Amplitude and Type of the Uplift of the Qinghai–Xizang Plateau.

[18] Qi D., Chao Y., Guo S., Zhao L., Li T., Wei F., Zhao X. (2012). Convergent, parallel and correlated evolution of trophic morphologies in the subfamily *Schizothoracinae* from the Qinghai-Tibetan plateau. PLoS ONE.

[19] Li Y., Luo Y., Lv Y., Ou Y., Zhang R., Zhang R. (2025). A Novel Mitochondrial Genome Resource for the Endemic Fish *Gymnodiptychus integrigymnatus* and Insights into the Phylogenetic Relationships of *Schizothoracinae*. Biology.

[20] Kousar Y., Singh M., Singh D. (2025). Morphometric and genetic insights into intraspecific variations in Himalayan snow trout (*Schizothorax richardsonii*) across major Indian Himalayan river drainages. J. Fish Biol..

[21] Ma B., Zhao T., Xu B., Zhong L., Wu X., Wei K., Zhang Z., Li Y. (2024). Morphological variation in *Schizothorax oconnori*, Schiz-*othorax waltoni* (Teleostei: *Cyprinidae*: *Schizothoracinae*), and their natural hybrids from the middle Yarlung Zangbo River, Tibet. Ecol. Evol..

[22] Cheng L., Song D., Yu X., Du X., Huo T. (2022). Endangered *Schizothoracin* Fish in the Tarim River Basin Are Threatened by In-trogressive Hybridization. Biology.

[23] Andersen M.M., Balding D.J. (2018). How many individuals share a mitochondrial genome?. PLoS Genet..

[24] DeRaad D.A., McCullough J.M., DeCicco L.H., Hime P.M., Joseph L., Andersen M.J., Moyle R.G. (2023). Mitonuclear discordance results from incomplete lineage sorting, with no detectable evidence for gene flow, in a rapid radiation of *Todiramphus* kingfishers. Mol. Ecol..

[25] Moran B.M., Payne C.Y., Powell D.L., Iverson E.N.K., Donny A.E., Banerjee S.M., Langdon Q.K., Gunn T.R., RodriguezSoto R.A., Madero A. (2024). A lethal mitonuclear incompatibility in complex I of natural hybrids. Nature.

[26] Payseur B.A., Rieseberg L.H. (2016). A genomic perspective on hybridization and speciation. Mol. Ecol..

[27] Hohenlohe P.A., Funk W.C., Rajora O.P. (2021). Population genomics for wildlife conservation and management. Mol. Ecol..

[28] Guo X.Z., Zhang G.R., Wei K.J., Yan R.J., Ji W., Yang R.B., Wei Q.W., Gardner J.P. (2016). Phylogeography and population genetics of *Schizothorax o’connori*: Strong subdivision in the Yarlung Tsangpo River inferred from mtDNA and microsatellite markers. Sci. Rep..

[29] Wang T., Qi D., Sun S., Liu Z., Du Y., Guo S., Ma J. (2019). DNA barcodes and their characteristic diagnostic sites analysis of *Schizothoracinae fishes* in Qinghai province. Mitochondrial DNA Part A.

[30] Sui X., Lin P., Ding Y., Wang H., Waters J., He D. (2026). Genetic insights into drainage evolution: Late Miocene river capture in the eastern Himalaya. J. Syst. Evol..

[31] Robinson R.A.J., Brezina C.A., Parrish R.R., Horstwood M.S.A., Oo N.W., Bird M.I., Thein M., Walters A.S., Oliver G.J.H., Zaw K. (2014). Large rivers and orogens: The evolution of the Yarlung Tsangpo–Irrawaddy system and the eastern Himalayan syntaxis. Gondwana Res..

[32] Rozimov A., Wang Y., Wang M., Zou M., Sobirov J., Karimov E., Kholmatov B., Freyhof J., Namozov S., Wang C. (2025). Mitochondrial genome insights into the phylogenetics and biogeographic evolution of snow trout (*Cyprinidae*, *Schizothorax*) in the Tien Shan Mountains. Zoosystematics Evol..

[33] Caves J.K., Bayshashov B.U., Zhamangara A., Ritch A.J., Ibarra D.E., Sjostrom D.J., Mix H.T., Winnick M.J., Chamberlain C.P. (2017). Late Miocene uplift of the Tian Shan and Altai and reorganization of Central Asia climate. GSA Today.

[34] Dai Y., Han H. (2018). Karyological analysis of two species in the subfamily *Schizothoracinae* (*Cypriniformes*: *Cyprinidae*) from China, with notes on karyotype evolution in *Schizothoracinae*. Turk. J. Fish. Aquat. Sci..

[35] Chen C.N., Huang Y.Y., Li H., Long Z.H., Lai J.S., Liu G.X., Zhao G. (2016). The complete mitochondrial genome of *Schizothorax prenanti* (Tchang) (*Teleostei*, *Cyprinidae*, *Schizothoracinae*). Mitochondrial DNA Part A.

[36] Gundappa M.K., To T.H., Grønvold L., Martin S.A.M., Lien S., Geist J., Hazlerigg D., Sandve S.R., Macqueen D.J. (2022). Genome-Wide Reconstruction of Rediploidization Following Autopolyploidization across One Hundred Million Years of Salmonid Evolution. Mol. Biol. Evol..

[37] Krabbenhoft T.J., MacGuigan D.J., Backenstose N.J.C., Hannah Waterman H., Lan T.Y., Pelosi J.A., Tan M., Sandve S.R. (2021). Chromosome-level genome assembly of *Chinese sucker* (*Myxocyprinus asiaticus*) reveals strongly conserved synteny following a catostomid-specific whole-genome duplication. Genome Biol. Evol..

[38] Li J.T., Wang Q., Huang Yang M.D., Li Q.S., Cui M.S., Dong Z.J., Wang H.W., Yu J.H., Zhao Y.J., Yang C.R. (2021). Parallel subgenome structure and divergent expression evolution of allo-tetraploid common carp and goldfish. Nat. Genet..

[39] Redmond A.K., Casey D., Gundappa M.K., Macqueen D.J., McLysaght A. (2023). Independent rediploidization masks shared whole genome duplication in the sturgeon-paddlefish ancestor. Nat. Commun..

[40] Lien S., Koop B.F., Sandve S.R., Miller J.R., Kent M.P., Nome T., Hvidsten T.R., Leong J.S., Minkley D.R., Zimin A. (2016). The Atlantic salmon genome provides insights into rediploidization. Nature.

[41] Sancho R., Inda L.A., Díaz-Pérez A., Des Marais D.L., Gordon S., Vogel J.P., Lusinska J., Hasterok R., Contreras-Moreira B., Catalán P. (2022). Tracking the ancestry of known and ‘ghost’ homeologous subgenomes in model grass Brachy-podium polyploids. Plant J..

[42] Yang L., Mayden R.L., Naylor G.J.P. (2025). Origin of Polyploidy, Phylogenetic Relationships, and Biogeography of Botiid Fishes (*Teleostei*: *Cypriniformes*). Biology.

[43] Glover N.M., Redestig H., Dessimoz C. (2016). Homoeologs: What Are They and How Do We Infer Them?. Trends Plant Sci..

[44] Chen L., Li C., Li B., Zhou X., Bai Y., Zou X., Zhou Z., He Q., Chen B., Wang M. (2024). Evolutionary divergence of subgenomes in common carp provides insights into speciation and allopolyploid success. Fundam. Res..

[45] Soares N.R., Mollinari M., Oliveira G.K., Pereira G.S., Vieira M.L.C. (2021). Meiosis in Polyploids and Implications for Genetic Mapping: A Review. Genes.

[46] Niu J., Zhang R., Hu J., Zhang T., Liu H., Minavar M., Zhang H., Xian W. (2022). Chromosomal-scale genome assembly of the near-extinction big-head schizothorcin (*Aspiorhynchus laticeps*). Sci. Data.

[47] Liang Y., He D., Jia Y., Sun H., Chen Y. (2017). Phylogeographic studies of *Schizothoracine* fishes on the central Qinghai-Tibet Plateau reveal the highest known glacial microrefugia. Sci. Rep..

[48] Lei L., Deng X., Liu F., Gao H., Duan Y., Li J., Fu S., Li H., Zhou Y., Liao R. (2024). Exploitation of Key Regulatory Modules and Genes for High-Salt Adaptation in *Schizothoracine* by Weighted Gene Co-Expression Network Analysis. Animals.

[49] Zhang J., Chen Z., Zhou C., Kong X. (2016). Molecular phylogeny of the subfamily *Schizothoracinae* (Teleostei: *Cypriniformes*: *Cy-prinidae*) inferred from complete mitochondrial genomes. Biochem. Syst. Ecol..

[50] Tong C., Fei T., Zhang C., Zhao K. (2017). Comprehensive transcriptomic analysis of Tibetan *Schizothoracinae* fish *Gymnocypris przewalskii* reveals how it adapts to a high altitude aquatic life. BMC Evol. Biol..

[51] Zhang D., Yu M., Hu P., Peng S., Liu Y., Li W., Wang C., He S., Zhai W., Xu Q. (2017). Genetic Adaptation of *Schizothoracine* Fish to the Phased Uplifting of the Qinghai-Tibetan Plateau. G3 Genes Genomes Genet..

[52] Chen Y., Wu X., Liu X., Lai J., Gong Q. (2023). Comparative transcriptome analysis provides insights into the TDG supersaturation stress response of *Schizothorax davidi*. Comp. Biochem. Physiol. Part C Toxicol. Pharmacol..

[53] Ban X., Dang Y., Shu P., Qi H., Luo Y., Xiao F., Feng Q., Zhou Y. (2024). Estimation of phytoplankton primary productivity in Qinghai Lake using ocean color satellite data: Seasonal and interannual variations. Water.

[54] Xu W., Zhu F., Wang D., Chen D., Duan X., Liu M., Li D. (2023). Comparative analysis of metabolites between different altitude *Schizothorax nukiangensis* (*Cyprinidae*, *Schizothoracine*) on the Qinghai-Tibet Plateau in Nujiang River. Water.

[55] Liu S., Tian F., Qi D., Qi H., Wang Y., Xu S., Zhao K. (2023). Physiological, metabolomic, and transcriptomic reveal metabolic pathway alterations in *Gymnocypris przewalskii* due to cold exposure. BMC Genom..

[56] Watanabe S., Overholt E.P., Schladow S.G., Vincent W.F., Williamson C.E. (2025). Climate change and underwater light: Large-scale changes in ultraviolet radiation transparency associated with intensifying wet–dry cycles. Limnol. Oceanogr. Lett..

[57] Qi D., Chao Y., Wu R., Xia M., Chen Q., Zheng Z. (2018). Transcriptome Analysis Provides Insights Into the Adaptive Responses to Hypoxia of a *Schizothoracine* Fish (*Gymnocypris eckloni*). Front. Physiol..

[58] Guan L., Chi W., Xiao W., Chen L., He S. (2014). Analysis of hypoxia-inducible factor alpha polyploidization reveals adaptation to Tibetan Plateau in the evolution of *Schizothoracine* fish. BMC Evol. Biol..

[59] Ma R., Chen Z., Wang B., Xu C., Jia Z., Li L., Hu J. (2024). Spatiotemporal Variations and Controlling Mechanism of Low Dis-solved Oxygen in a Highly Urbanized Complex River System. J. Hydrol. Reg. Stud..

[60] Benson A., Zane M., Becker T.E., Visser A., Uriostegui S.H., DeRubeis E., Moran J.E., Esser B.K., Clark J.F. (2014). Quantifying reaeration rates in alpine streams using deliberate gas tracer experiments. Water.

[61] Liang Y., Hou Y., Hu W., Johnson D., Wang J. (2021). Flow velocity preference of *Schizothorax oconnori* Lloyd swimming upstream. Glob. Ecol. Conserv..

[62] Reemeyer J.E., Chapman L.J. (2024). Effects of Acute Hypoxia Exposure and Acclimation on the Thermal Tolerance of an Imperiled Canadian Minnow. J. Exp. Zool. A Ecol. Integr. Physiol..

[63] Mucha S., Chapman L.J., Krahe R. (2023). Normoxia exposure reduces hemoglobin concentration and gill size in a hypoxia-tolerant tropical freshwater fish. Environ. Biol. Fishes.

[64] Jin Y., Zhu B., Wang F., Sun S., Wang P., Liu X. (2023). Analysis of water chemistry characteristics and main ion controlling factors of lakes in the Nagqu area of the Qinghai–Tibet plateau in summer. Water.

[65] Tong C., Li M. (2020). Genomic signature of accelerated evolution in a saline-alkaline lake-dwelling *Schizothoracine* fish. Int. J. Biol. Macromol..

[66] Kousar Y., Singh M., Singh D. (2025). Stock assessment of *Schizothorax richardsonii* (Gray, 1832) using geometric morphometrics and mitochondrial marker COX1 from tributaries of the Chenab River, India. J. Appl. Nat. Sci..

[67] Gul S., Rashid I. (2025). Morphological variations among *Schizothorax* species from Kashmir Himalayas. Zool. Anz..

[68] Rajput V., Johnson J.A., Sivakumar K. (2013). Environmental effects on the morphology of the snow trout *Schizothorax richardsonii* (Gray, 1832). TAPROBANICA J. Asian Biodivers..

[69] Martinez-Leiva L., Landeira J.M., Fatira E., Díaz-Pérez J., Hernández-León S., Roo J., Tuset V.M. (2023). Energetic Implications of Morphological Changes between Fish Larval and Juvenile Stages Using Geometric Morphometrics of Body Shape. Animals.

[70] Reyes Corral W.D., Aguirre W.E. (2019). Effects of temperature and water turbulence on vertebral number and body shape in Astyanax mexicanus (*Teleostei*: *Characidae*). PLoS ONE.

[71] Zhou L., Jin Z.D., Li F.C. (2012). Mineralogy of the otoliths of naked carp *Gymnocypris przewalskii* (Kessler) from Lake Qinghai and its Sr/Ca potential implications for migratory pattern. Sci. China Earth Sci..

[72] Belay T.H., Mengist A.B. (2025). The application of otolith chemistry in fish life history assessment. Fish. Aquat. Sci..

[73] Zhou Y., He Z., Cui W., Lu Q., Qin J., Han Z., Liu J., He T. (2025). Strontium and Magnesium in Otoliths Can Trace Schizothorax grahami (Regan, 1904) Life History. Animals.

[74] Kratina G.J., DeVries D.R., Wright R.A., Peatman E., Rider S.J., Zhao H. (2023). Using fish hard-part microchemistry and genetics to quantify population impacts of low-use lock-and-dam structures on the Alabama River. Trans. Am. Fish. Soc..

[75] Wang G., Tang Q., Chen Z., Guo D., Zhou L., Lai H., Li G. (2022). Otolith microchemistry and demographic history provide new insight into the migratory behavior and heterogeneous genetic divergence of coilia grayii in the pearl river. Fishes.

[76] Sturrock A.M., Hunter E., Milton J.A., Johnson R.C., Waring C.P., Trueman C.N., EIMF (2015). Quantifying physiological influ-ences on otolith microchemistry. Methods Ecol. Evol..

[77] Ma B., Xie C., Huo B., Yang X., Huang H. (2010). Age and growth of a long-lived fish *Schizothorax o’connori* in the Yarlung Tsangpo River, Tibet. Zool. Stud..

[78] Ma B., Xie C., Huo B., Yang X., Li P. (2011). Age validation, and comparison of otolith, vertebra and opercular bone for estimating age of *Schizothorax o’connori* in the Yarlung Tsangpo River, Tibet. Environ. Biol. Fishes.

[79] Huo B., Ma B.S., Xie C.X., Duan Y.J., Yang X.F., Huang H.P. (2015). Stock assessment and management implications of an endemic fish, *Oxygymnocypris stewartii*, in the Yarlung Zangbo River in Tibet, China. Zool. Stud..

[80] Pfennig M.B., Crane D.P., Smith N.G., Buckmeier D.L. (2024). Age estimation and validation in otoliths, spines, and fin rays from four central Texas fishes. N. Am. J. Fish. Manag..

[81] Xu L., Zhou Y., Cui W., Lu Q., Liu J., Duan C., He T. (2024). Characteristics of surface flow field and substrate in the spawning ground of *Schizothorax grahami*. Isr. J. Aquac..

[82] Pan H., Liu H., Liu F., Xie J., Zhou Y., Zheng Q., Guo M. (2025). Gut microbiota: A new frontier in understanding and protecting endangered plateau *Schizothorax* fish. Front Microbiol..

[83] Yan T., Liu F., Chang M., Yan R., Luo W., Wen L., Ding W., Fu Q., Wang X., Yang D. (2025). Morphological Differences in Feeding and Digestive Organs, the Diversity of Intestinal Microorganisms, and Variations in Digestive Enzyme Activity Promote the Differentiation of Nutritional Niches in *Schizothoracinae* Species. Animals.

[84] Wang X., Hao J., Zhang C., Zhu P., Gao Q., Liu D., Nie M., Jia J., Qi D. (2025). Differences and Correlation Analysis of Feeding Habits and Intestinal Microbiome in *Schizopygopsis microcephalus* and *Ptychobarbus kaznakovi* in the Upper Reaches of Yangtze River. Front. Microbiol..

[85] Zhong C., Chen L., Huang Z., Hu Y., Jiang Y., Zhou J., Long X. (2024). Comparison of metabolism, gut histology, and microbiota between *Schizothorax lissolabiatus* and *Schizothorax griseus* under identical farming conditions. Front. Mar. Sci..

[86] Mamoozadeh N.R., Wade M.J., Reid B.N., Bardwell E., Collins E.E., Hugentobler S.A., Jackson S.A., Kline B.C., Rothkopf H.E., Zhang A. (2025). A practical introduction to effective population size for fisheries management. Trans. Am. Fish. Soc..

[87] Funk W.C., McKay J.K., Hohenlohe P.A., Allendorf F.W. (2012). Harnessing genomics for delineating conservation units. Trends Ecol. Evol..

[88] Semlitsch R.D., Hotz H., Guex G.D. (1997). Competition among tadpoles of coexisting hemiclones of hybridogenetic Rana esculenta: Support for the frozen niche variation model. Evolution.

[89] Liao J., Chen S., Liu P., Fontaneto D., Han B.P. (2024). Environmental selection and gene flow jointly determine the population genetic diversity and structure of *Diaphanosoma dubium* along a watershed elevation. Glob. Ecol. Conserv..

[90] Cooke S.J., Hinch S.G. (2013). Improving the reliability of fishway attraction and passage efficiency estimates to inform fishway engineering, science, and practice. Ecol. Eng..

[91] Laikre L., Schwartz M.K., Waples R.S., Ryman N., GeM Working Group (2010). Compromising genetic diversity in the wild: Un-monitored large-scale release of plants and animals. Trends Ecol. Evol..

[92] Bouwmeester M.M., Goedknegt M.A., Poulin R., Thieltges D.W. (2021). Collateral diseases: Aquaculture impacts on wildlife infections. J. Appl. Ecol..

[93] Wang F., Wang L., Liu D., Gao Q., Nie M., Zhu S., Chao Y., Yang C., Zhang C., Yi R. (2022). Chromosome-level assembly of *Gymnocypris ecklonigenome*. Sci. Data.

[94] Wang Q., Lan T., Li H., Sahu S.K., Shi M., Zhu Y., Han L., Yang S., Li Q., Zhang L. (2022). Whole-genome resequencing of Chinese pangolins reveals a population structure and provides insights into their conservation. Commun. Biol..

[95] He J., He Z., Yang D., Ma Z., Chen H., Zhang Q., Deng F., Ye L., Pu Y., Zhang M. (2022). Genetic Variation in *Schizothorax kozlovi Nikolsky* in the Upper Reaches of the Chinese Yangtze River Based on Genotyping for Simplified Genome Sequencing. Animals.

[96] Wedekind C. (2002). Sexual selection and life-history decisions: Implications for supportive breeding and the management of captive populations. Conserv. Biol..

[97] Han H., Wang L., Zhang C., Li H., Ma B. (2025). Population Structure, Growth Characteristics, Resource Dynamics, and Management Strategies of *Schizopygopsis younghusbandi* in Four Tributaries of the Yarlung Zangbo River, Tibet. Biology.

[98] Hixon M.A., Johnson D.W., Sogard S.M. (2014). BOFFFFs: On the importance of conserving old-growth age structure in fishery populations. ICES J. Mar. Sci..

[99] Bunt C.M., Castro-Santos T., Haro A. (2012). Performance of fish passage structures at upstream barriers to migration. River Res. Appl..

[100] Humphries P., King A., McCasker N., Kopf R.K., Stoffels R., Zampatti B., Price A. (2020). Riverscape recruitment: A conceptual synthesis of drivers of fish recruitment in rivers. Can. J. Fish. Aquat. Sci..

[101] King A.J., Gwinn D.C., Tonkin Z., Mahoney J., Raymond S., Beesley L. (2016). Using abiotic drivers of fish spawning to in-form environmental flow management. J. Appl. Ecol..

[102] Krellwitz E.M., Gido K.B., Mehl H.E., Totten L.A., Jones T.C. (2026). Abiotic Drivers of Spawning and Early Life Stage Assemblage Dynamics of Great Plains Fishes. River Res. Appl..

[103] Frankham R., Ballou J.D., Eldridge M.D., Lacy R.C., Ralls K., Dudash M.R., Fenster C.B. (2011). Predicting the probability of outbreeding depression. Conserv. Biol..

[104] Xu Y., Li J., Chang L., An R. (2025). Climate-Induced Changes in Habitat Suitability for a Cold-Water Endemic Species in the Lancang River Basin: A Case Study of *Schizothorax lantsangensis*. Ecol. Evol..

[105] Nie M.A., Schultz C.A. (2012). Decision-making triggers in adaptive management. Conserv. Biol..

[106] Palmé A. (2010). Assessing and Monitoring Genetic Patterns for Conservation Purposes with Special Emphasis on Scandinavia. Ph.D. Thesis.

[107] Santos J.M., Quaresma A.L., Romão F., Amaral S.D., Mameri D., Santo M., Bochechas J., Telhado A., Godinho F.N., Pádua J. (2025). Fishways in Portugal: Status, Main Findings and Research Priorities. Water.

[108] Martínez-Páramo S., Horváth Á., Labbé C., Zhang T., Robles V., Herráez P., Suquet M., Adams S., Viveiros A., Tiersch T.R. (2017). Cryobanking of aquatic species. Aquaculture.

[109] Liu Q.Y., Li J., An R.D., Li Y. (2018). Ecohydraulogical characteristic index system of *Schizopygopsis younghusbandi* during spawning periods in the Yarlung Tsangpo River. Int. J. Environ. Res. Public Health.

